# Functional exercise, fear of recurrence, and sleep quality: a longitudinal path analysis of quality of life in postoperative breast cancer patients

**DOI:** 10.3389/fonc.2026.1607256

**Published:** 2026-03-11

**Authors:** Yujie Fei, Fenghe Liu, Xinru Ding, Jingyi Tang, Xu Ye, Tianhao Zhou, Haiping Xu

**Affiliations:** School of Nursing, Nanjing Medical University, Nanjing, China

**Keywords:** breast cancer, fear of cancer recurrence, functional exercise, longitudinal study, path analysis, Quality of Life, sleep quality

## Abstract

**Objective:**

This study aims to investigate the impacts of functional exercise, fear of cancer recurrence (FCR), and sleep quality on the quality of life (QoL) of postoperative breast cancer patients and to elucidate their interrelationships via path analysis.

**Methods:**

A total of 50 eligible breast cancer patients were enrolled via convenience sampling (first diagnosis of primary breast cancer, age ≥18 years, modified radical mastectomy, normal communication and understanding ability, ability of patients or their family members to use electronic devices). The Functional Assessment of Cancer Therapy—Breast (FACT-B), Postoperative Functional Exercise Compliance Scale, Fear of Cancer Recurrence Inventory—Short Form (FCRI-SF), and Pittsburgh Sleep Quality Index (PSQI) were used to assess participants at three time points: pre-discharge (T1), 1 month post-surgery (T2), and 3 months post-surgery (T3). Four participants were lost to follow-up (attrition rate of 7.4%). Missing data from participant attrition were handled using generalized estimating equations (GEE), which yield valid inferences under the missing completely at random (MCAR) assumption.

**Results:**

The QoL scores fluctuated significantly over time (T1: 87.44 ± 11.32, T2: 104.16 ± 8.94, T3: 100.50 ± 11.02). GEE analysis identified that older age (*β* = -12.35, 95% CI: -19.18 to -5.52), higher BMI (e.g., normal weight: *β* = -17.46, 95% CI: -28.67 to -6.26), longer daily working hours (e.g., 6–10 h/day: *β* = -11.86, 95% CI: -16.30 to -7.43), and slower daily life pace (e.g., slow: *β* = -8.93, 95% CI: -14.51 to -3.36) were significant negative predictors of QoL (all *P* < 0.05). The path analysis revealed that FCR exerted a significant direct negative effect on QoL (*β* = -0.920, *P* < 0.001) and an indirect effect by reducing exercise compliance (indirect effect: -0.166). Poor sleep quality (PSQI score) directly impaired QoL (*β* = -1.139, *P* = 0.003), while functional exercise compliance had a marginally significant positive direct effect (*β* = 0.874, *P* = 0.052).

**Conclusion:**

This exploratory study found that functional exercise, FCR, and sleep quality are dynamic and interrelated factors that significantly influence QoL after breast cancer surgery in the studied sample. Clinicians should implement staged, personalized interventions targeting these modifiable factors to improve postoperative patient outcomes.

## 1 Introduction

Breast cancer remains one of the most common malignancies affecting women worldwide. While advances in diagnosis and treatment have significantly improved the survival rates (1, 2), conventional therapies such as surgery, radiotherapy, and chemotherapy often impose considerable physical and psychological burdens on patients, which adversely impair their postoperative quality of life (QoL) (3). Accumulating evidence suggests that exercise, psychological state, and sleep play crucial roles in improving QoL among breast cancer survivors (4–6). Physical activity has been demonstrated to alleviate fatigue, anxiety, and depression as well as to enhance sleep quality and overall well-being (6). Importantly, adequate sleep may further promote exercise adherence (6), which indicates potential synergistic effects among these factors.

These relationships can be interpreted through complementary theoretical frameworks. The fear of cancer recurrence model (FCR) posits that fear of recurrence triggers intrusive thoughts and hypervigilance, which may lead to maladaptive coping behaviors (e.g., activity avoidance including exercise) and sleep disturbances, ultimately impairing QOL (7, 8). Concurrently, from the perspective of exercise oncology, regular physical activity is theorized to improve QoL through multiple pathways (1): by enhancing physical function and reducing treatment-side effects (2), by modulating psychological distress through neurobiological mechanisms (e.g., endorphin release and reduced inflammation), and (3) by improving sleep patterns (9–11). In this study, functional exercise refers specifically to the standardized postoperative rehabilitation exercises for breast cancer patients, as outlined by Lu (12), which is consistent with mainstream clinical guidelines.

However, longitudinal studies integrating these theoretical frameworks to explore their dynamic interactions are still scarce. Most existing evidence is cross-sectional, thus limiting the understanding of temporal relationships and potential causal mechanisms. Building on these frameworks, this study adopted a longitudinal design to explore how FCR may impair the beneficial effects of exercise by reducing adherence to postoperative functional exercise (standardized rehabilitation per guidelines (12)). We hypothesize the following: (1) FCR exerts a significant direct negative effect on QoL, (2) FCR indirectly impairs QoL through its negative effects on functional exercise adherence (i.e., reduced adherence mediates the FCR-QoL association), (3) FCR indirectly impairs QoL through its negative effects on sleep quality, and (4) functional exercise adherence exerts a direct positive effect on QoL, and this effect is attenuated by high levels of FCR. This study further analyzes the synergistic effects of exercise, psychological state, and sleep, aiming to provide a scientific basis for the development of personalized intervention strategies.

## 2 Subjects and methods

### 2.1 Respondents

The sample size of 50 was determined based on data availability, recruitment feasibility within the study period, and the exploratory nature of this preliminary investigation. A *post-hoc* power analysis was performed with G*Power (version 3.1) for multiple regression (*α* = 0.05, effect size *f*² = 0.25), which indicated a power of 0.78 to detect medium effects—a value considered acceptable for exploratory longitudinal studies. While a formal power analysis suggested a larger optimal sample size for path analysis, our sample size is comparable to that of similar longitudinal pilot studies and yields preliminary insights into the dynamic relationships examined in this study (13, 14).

The inclusion criteria were as follows: (1) first diagnosis of breast cancer, (2) aged ≥18 years, (3) underwent modified radical mastectomy for breast cancer, (4) normal communication and comprehension abilities, and (5) ability to use electronic devices (e.g., computers, mobile phones) for Internet access (patients or their family members). The exclusion criteria were as follows: (1) combined with other cancers, (2) cancer recurrence or metastasis, and (3) severe cardiopulmonary, hepatic, or renal dysfunction or end-stage disease. This study was conducted in accordance with the Declaration of Helsinki and was approved by the Ethics Committee of The First Affiliated Hospital of Nanjing Medical University (approval no.: 2023-SR-770). Written informed consent was obtained from all participants prior to data collection.

### 2.2 Survey tools

#### 2.2.1 General information questionnaire

The General Information Questionnaire, developed specifically for this study, was used to collect key demographic and clinical characteristics. Its content validity was evaluated via a literature review on factors influencing QoL in breast cancer survivors and expert assessment by a panel of five experts (oncologists, clinical nurses, and an epidemiologist). Items with a content validity index (CVI) score <0.78 were revised or eliminated. The final version of the questionnaire included core variables such as age, BMI, marital status, occupational status, cancer stage, treatment modality, and exercise behavior, thus ensuring clinical relevance and methodological rigor.

#### 2.2.2 Postoperative functional exercise compliance scale for breast cancer patients

The scale was developed by Lu (12) base on an extensive body of research evidence (15). The scale contains 18 items and uses a four-point Likert scale. Total scores range from 18 to 72, with higher scores representing better adherence to functional exercise regimens. The scale comprises three dimensions. Cronbach’s *α* coefficients for the total scale and the three dimensions were 0.899, 0.790, and 0.754, respectively, indicating good internal consistency reliability.

#### 2.2.3 Fear of Cancer Recurrence Inventory—Short Form

The Fear of Cancer Recurrence Inventory—Short Form (FCRI-SF) was derived from the Fear of Cancer Recurrence Inventory (FCRI), which exhibits good internal consistency (Cronbach’s *α* = 0.89) and test–retest reliability. The FCRI comprises seven dimensions (8): psychological distress, triggering factors, coping strategies, self-perception, functional impairment, reassurance, and severity. The severity dimension is highly correlated with the total FCRI score. Thus, the FCRI-SF scale was used to assess the level of FCR in patients. This scale uses a 0–4 point rating scale, consists of nine items, and has a total score ranging from 0 to 36. Higher total scores indicate a higher level of FCR. Individuals with an FCRI score of or exceeding 13 are categorized as having a high level of fear of cancer recurrence (16).

#### 2.2.4 Pittsburgh Sleep Quality Index

The Pittsburgh Sleep Quality Index (PSQI) was developed by Buysse et al. (17) in 1989 to assess sleep quality and sleep disturbances in adults. The PSQI consists of 19 items across seven dimensions, with each dimension scored on a 0–3 point scale. Item scores are summed to yield a total score ranging from 0 to 21, with higher scores indicating poorer sleep quality (18). The scoring criteria were as follows: (1) sleep quality: scored based on responses to item 6, (2) sleep latency: sum of item 2 and item 5a scores, with a total score of 0 assigned zero points, 1–2 assigned one point, 3–4 assigned two points, and 5–6 assigned three points, (3) total sleep time: scored based on responses to item 4, (4) sleep efficiency: calculated as total sleep time/bedtime, with scores assigned by efficiency range: >85% = 0 points, 75%–84% = 1 point, 65%–74% = 2 points, <65% = 3 points, (5) sleep disturbances: sum of scores from items 5b to 5i, with a total score of 0 assigned zero points, 1–9 assigned one point, 10–18 assigned two points, and 19–27 assigned three points, (6) use of sleeping medication: scored based on responses to item 7, and (7) daytime dysfunction: sum of scores from items 8 and 9, with a total score of 0 assigned zero points, 1–2 assigned one point, 3–4 assigned two points, and 5–6 assigned three points.

#### 2.2.5 Functional Assessment of Cancer Therapy—Breast

The Functional Assessment of Cancer Therapy—Breast (FACT-B) was developed by Cella et al. at the US Medical Research Center and later translated into Chinese by Wan et al. (19) in 2002. The Chinese version of the FACT-B was used to assess QoL in patients with breast cancer. The scale consists of 36 items across five dimensions. Scores for each dimension are summed to yield a total score, with higher scores indicating better QoL (20).

### 2.3 Date collection methods

This prospective longitudinal study was conducted in the Breast Surgery Wards of a university-affiliated grade III class A hospital. The participants were recruited postoperatively after their condition had stabilized (usually on postoperative days 2 to 3). Trained research staff explained the study purpose, procedures, potential risks, and benefits to the participants and emphasized to them that participation was voluntary. Written informed consent was obtained from all patients who agreed to participate.

Data were collected electronically via Questionnaire Star platform at three time points: pre-discharge (T1, 3 to 4 days post-surgery), 1 month post-surgery (T2), and 3 months post-surgery (T3). T1 assessment was completed in the hospital using a tablet device, while T2 and T3 assessments were conducted via encrypted electronic links sent to participants’ mobile phones following a reminder call with brief guidance. Brief verbal guidance was provided before each follow-up assessment to ensure data quality.

To minimize potential selection bias, data collection was conducted by trained research staff who were blinded to the study hypotheses and not involved in the clinical care of the participants. All participants were assigned a unique identification code to ensure anonymity during data collection and analysis.

At T1, general demographic characteristics, QoL, functional exercise compliance, FCR level, and PSQI score were collected. The same indicators were reassessed at T2 and T3. A total of 54, 52, and 50 participants were included in the T1, T2, and T3 assessments, respectively. Four participants were lost to follow-up (two declined to answer phone calls and two withdrew from the study), resulting in an effective follow-up rate of 92.6%.

### 2.4 Statistical analyses

Statistical analysis was performed using SPSS 26.0 and Amos software. Categorical variables were described as frequencies and percentages. Normally distributed continuous variables were expressed as mean ± standard deviation (SD), while non-normally distributed continuous variables were summarized as median (25th, 75th percentiles). Scores for QoL, functional exercise compliance, FCR, and PSQI were normally distributed at all three time points. The assumption of sphericity was violated for functional exercise compliance scores, and the Greenhouse–Geisser correction was applied for subsequent analyses. Repeated-measures analysis of variance (ANOVA) was used to analyze within-group changes across the three time points.

Generalized estimating equations (GEE) were used to identify factors influencing quality of life, accounting for within-subject correlations in longitudinal data. An unstructured correlation matrix was used for flexibility. The model included time, repeated measures (exercise adherence, sleep quality, and FCR level), and time-invariant covariates (demographic characteristics, cancer stage, and adjuvant therapy). Multicollinearity was assessed using variance inflation factors (VIFs), with a VIF <10 indicating no significant multicollinearity. Model fit was evaluated using the quasi-likelihood under the independence model criterion (QIC). Missing data from the four lost-to-follow-up cases were handled under the missing completely at random (MCAR) assumption within GEE, which provides unbiased estimates under MCAR.

Structural equation modeling (path analysis) was chosen as it allows for the simultaneous testing of multiple direct and indirect effects, which aligns with our study’s aim to examine the complex mediation relationships among FCR, exercise adherence, sleep, and QoL. Path analysis was conducted in Amos using FIML estimation, robust under MAR assumptions. Model fit was assessed using the chi-square/degrees of freedom ratio (*χ*²/df), goodness-of-fit index (GFI), comparative fit index (CFI), normative fit index (NFI), and root mean square error of approximation (RMSEA). The robustness of the path coefficients was evaluated using the bias-corrected bootstrap method with 1,000 resamples, which is recommended for assessing stability in samples of modest size. A *p*-value <0.05 indicated statistical significance.

## 3 Results

### 3.1 Demographic and clinical characteristics of participants

A total of 50 patients were included in this study and completed all three longitudinal assessments. The demographic details of the participants are outlined in **Table 1**.

**Table 1:** Demographic and clinical characteristics of the study participants (*n* = 50).

Items	*n* (%)	Items	Number of cases (percentage, %)
Age (years)		Professional status	
18–35	3 (6.0)	Unemployed	7 (14.0)
36–59	31 (62.0)	Retiring	14 (28.0)
≥60	16 (32.0)	On the job	29 (58.0)
BMI		Daily working hours (h/day)	
<18.5 (underweight)	1 (2.0)	≤6	12 (24.0)
18.5–24.9 (normal weight)	33 (66.0)	6-10	36 (72.0)
25–29.99 (overweight)	14 (28.0)	>10	1(2.0)
≥30 (obese)	2 (4.0)	Unfixed	1(2.0)
Cancer stage		Treatment modalities	
II	34 (68.0)	Radiotherapy	29 (58.0)
III	16 (32.0)	Chemotherapy	31 (62.0)
Marital status		Children’s status	
Married	45 (90.0)	Yes	46 (92.0)
Unmarried	5 (10.0)	No	4 (8.0)
Overtime work		Work stress	
Yes	9 (18.0)	Big	13 (26.0)
No	41 (82.0)	Small	17 (34.0)
Daily life pace		No	20 (40.0)
Fast	14 (28.0)	Daily chores	
Medium	23 (46.0)	Yes	37 (74.0)
Slow	13 (26.0)	No	13 (26.0)
Daily steps		Exercise intensity	
≤5,000	15 (30.0)	Light exercise	37 (74.0)
5,001–10,000	23 (46.0)	Low-intensity exercise	6 (12.0)
10,001–15,000	10 (20.0)	Moderate intensity exercise	7 (14.0)
≥15,001	2 (4.0)		
Past history		Family history	
Yes	17 (34.0)	Yes	6 (12.0)
No	33 (66.0)	No	44 (88.0)
History of allergies		History of smoking and drinking	
Yes	6 (12.0)	Yes	4 (8.0)
No	44 (88.0)	No	46 (92.0)
Time to exercise		Frequency of exercise	
≤10min	15 (30.0)	Once a month	9 (18.0)
11–20 min	8 (16.0)	2–3 times a month	10 (20.0)
21–30 min	9 (18.0)	1 or 2 times a week	15 (30.0)
31–59 min	7 (14.0)	3–5 times a week	9 (18.0)
≥60 min	11 (22.0)	1 times a day	7 (14.0)

Data on clinical characteristics including cancer stage (II: *n* = 34, III: *n* = 16) and treatment modalities (chemotherapy: *n* = 31, radiotherapy: *n* = 29) were also collected but are not presented here due to limited variability in this cohort.

### 3.2 Comparisons of core outcome scores across three time points

The results showed that patients had the lowest QoL and highest FCR scores at T1, indicating the poorest QoL and the most severe psychological distress related to FCR at this time point. The QoL and FCR scores differed significantly across the three time points (all *P* < 0.05). Patients had the highest functional exercise compliance and the lowest PSQI scores (best sleep quality) at T2 (**Table 2**).

**Table 2:** Breast cancer patients with different time scale score comparison (*n* = 50).

Items	T1	T2	T3	*F*	*P*	Multiple comparisons
Quality of life	87.44 ± 11.32	104.16 ± 8.94	100.5 ± 11.02	40.43	0.000	Ab
Functional exercise adherence	58.56 ± 6.83	68.94 ± 3.22	67.48 ± 2.71	76.49	0.000	abc
Fear of cancer recurrence	19.34 ± 3.80	15.38 ± 4.65	12.66 ± 4.89	28.25	0.000	abc
Pittsburgh Sleep Quality Index	8.90 ± 3.36	5.90 ± 3.35	7.34 ± 3.09	11.54	0.000	abc

a, T1 is significantly different from T2; b, significant difference between T1 and T3; c, T2 is significantly different from T3.

### 3.3 Analysis of the influencing factors of quality of life in breast cancer patients

The quality of life scores of patients was considered the dependent variable, while general demographic information, as well as the functional exercise adherence of patients (T1–T3), Pittsburgh Sleep Quality Index, and fear of cancer recurrence score were considered the independent variables; all variables were incorporated into a generalized estimating equations model. Variable assignment is shown in **Table 3**. The results of the study showed that factors such as age, BMI, occupational status, daily working hours, daily pace, exercise intensity, exercise time, functional exercise adherence, fear of cancer recurrence, and the Pittsburgh Sleep Quality Index influenced the quality of life for patients. It is noteworthy that all sociodemographic and clinical variables listed in **Table 3** were included in the generalized estimating equation model. However, to enhance the clarity and focus of the results, **Table 4** presents only the variables that demonstrated a statistically significant association with quality of life (*P* < 0.05). Multicollinearity diagnostics confirmed that all time-invariant covariates had variance inflation factor (VIF) values well below the threshold of 10 (range: [1.252 to 3.972]), indicating that multicollinearity was not a substantial concern in the model. The results are shown in **Table 4**.

**Table 3:** Assignment of variables.

Variable	Assignment
Quality of life	Original value input
Functional exercise compliance	Original value input
Pittsburgh Sleep Index	Original value input
Fear of cancer recurrence	Original value input
Age	Youth: age 18–35 = 1; middle: age 36–59 = 2; old: age 60 = 3
BMI	<18.5 underweight = 1; 18.5–24.9 normal weight = 2; 25-29.9 overweight = 3; ≥30 obesity = 4
Professional status	Jobless =1; retired = 2; on-the-job = 3
Work time every day	≤6 h/day = 1; 6–10 h = 2; >10 h = 3; unfixed = 4
Need to work overtime	Yes = 1; no = 2
Work pressure	Big = 1; small = 2; no = 3
Marital status	Married = 1; unmarried = 2
Child status	Yes = 1; no = 2
Previous history	Yes = 1; no = 2
Family history	Yes = 1; no = 2
History of allergies	Yes = 1; no = 2
History of smoking and drinking	Yes = 1; no = 2
Daily pace	Fast = 1; medium = 2; slow = 3
Daily steps	<5,000 = 1; 5,000–10,000 = 2; 10,000–15,000 = 3; >15,000 = 4
Daily chores	Yes = 1; no = 2
Intensity of exercise	Slight movement = 1; light-intensity exercise = 2; moderate intensity = 3
Time to exercise	Under 10 min = 1; 11–20 min = 2; 21–30 min = 3; 31–59 min = 4; more than 60 min = 5
Exercise frequency	Once a month = 1; 2–3 times a month = 2; 1 or 2 times a week = 3; 3–5 times a week = 4; once a day = 5

**Table 4:** Generalized estimating equation analysis of factors influencing quality of life in breast cancer patients (*n* = 50).

Variable	*β*	SE	Wald	*P*	95% CI
Agea					
Middle age 36–59	-0.521	2.7528	0.036	0.850	[-5.917, 4.874]
Old age ≥60 years BMIb	-12.350	3.4848	12.560	0.000	[-19.180, -5.520]
18.5–24.9 normal weight	-17.464	5.7177	9.330	0.002	[-28.671, -6.258]
25–29.9 overweight	-11.777	4.9918	5.566	0.018	[-21.561, -1.993]
≥30 obese	-3.517	7.4279	0.224	0.636	[-18.075, 11.042]
Career statusc					
Retiring	13.624	2.9117	21.893	0.000	[7.917, 19.331]
On-the-job	6.002	3.3155	3.277	0.070	[-.496, 12.500]
Hours worked per dayd					
6–10 h	-11.863	2.2639	27.459	0.000	[-16.300, -7.426]
>10 h	-3.844	3.9584	0.943	0.332	[-11.602, 3.915]
Unfixed	-16.564	5.2108	10.104	0.001	[-26.777, -6.351]
Daily pacee					
Medium	-6.071	2.2081	7.559	0.006	[-10.399, -1.743]
Slow	-8.934	2.8455	9.858	0.002	[-14.511–3.357]
Intensity of exercisef					
Light intensity exercise	3.963	2.0895	3.596	0.058	[-0.133, 8.058]
Moderate intensity	12.281	2.2034	31.065	0.000	[7.962, 16.600]
Exercise timeg (min)					
11–20	-5.156	2.2407	5.296	0.021	[-9.548, -0.765]
21–30	-6.199	3.4684	3.194	0.074	[-12.997, 0.599]
31–59	-2.148	3.1952	0.452	0.502	[-8.410, 4.115]
≥60	-4.342	3.4297	1.603	0.205	[-11.065, 2.380]
Functional exercise adherence	0.641	0.1240	26.706	0.000	[0.398, 0.884]
Fear of cancer recurrence	-0.753	0.1666	20.452	0.000	[-1.080, -0.427]
Pittsburgh Sleep Index	-1.584	0.2004	62.435	0.000	[-1.976, -1. 191]
Scale	56.169				

a 18–36 years old as reference.

b <18.5 as reference.

c Unemployed as reference.

d <6 h as reference.

e Fast as reference.

f Mild exercise for reference.

g <10 min as reference.

### 3.4 Path analysis of functional exercise, fear of cancer recurrence, and sleep on patients’ quality of life

**Figure 1:**
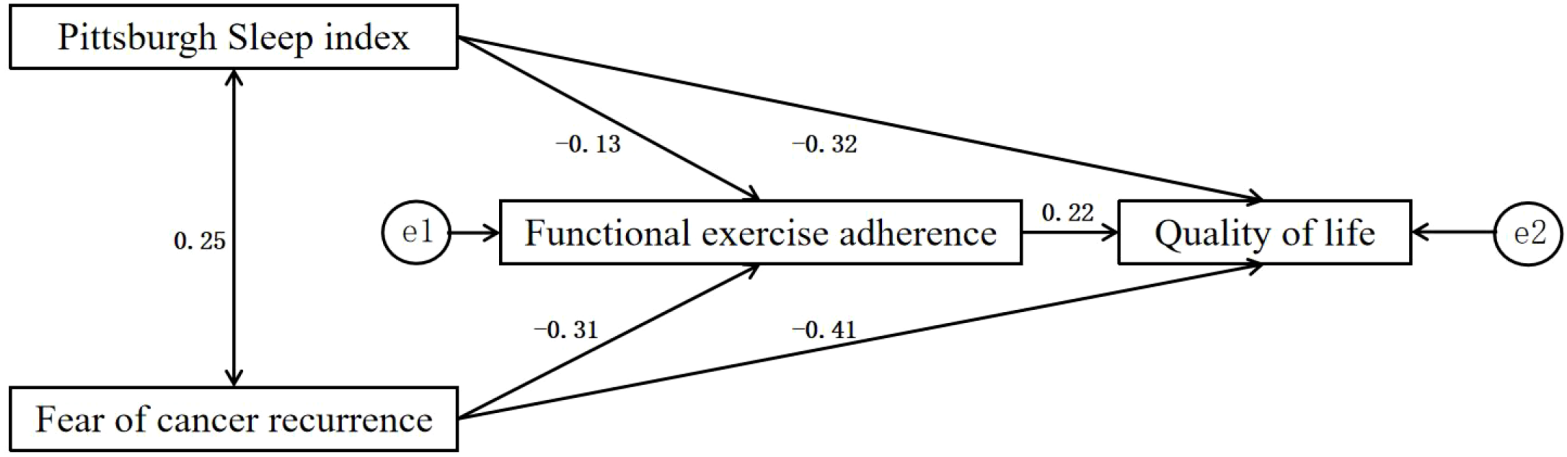
Original path mode.

**Figure 2:**
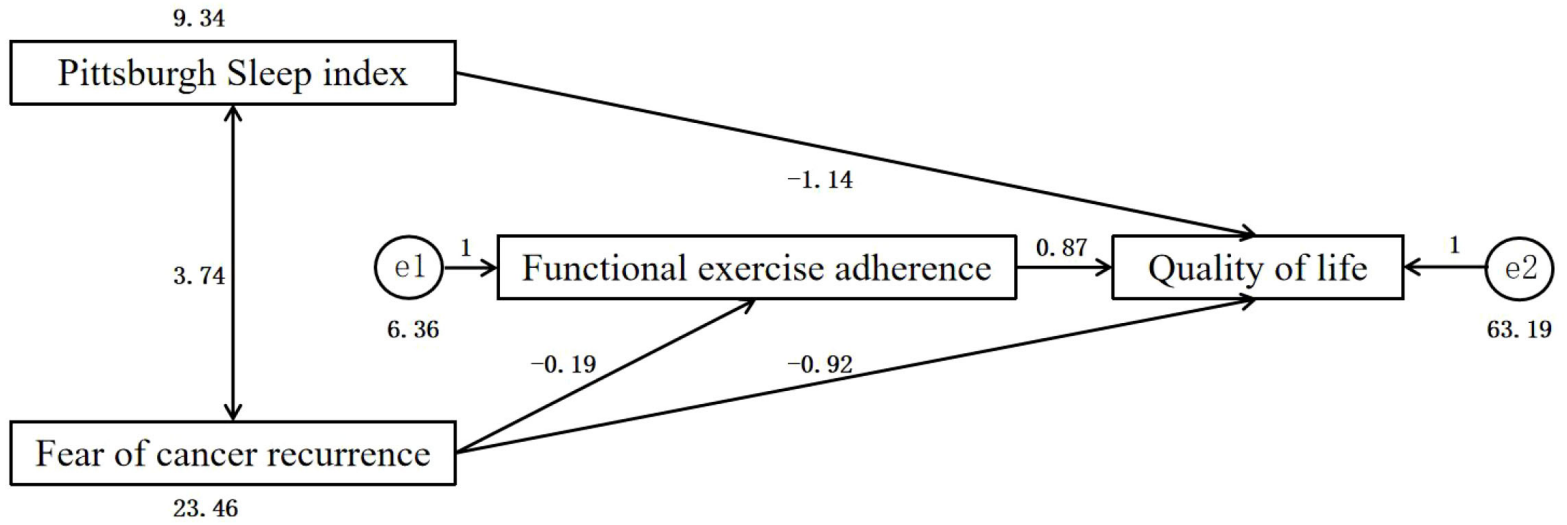
Path analysis of the quality of life of breast cancer patients.

This study adopted structural equation modeling to explore the determinants of quality of life at T3, with functional exercise compliance, fear of cancer recurrence, and the Pittsburgh Sleep Quality Index as exogenous variables. The initial saturated model (*χ*² = 0, df = 0) was not suitable for model fit evaluation (**Figure 1**). After removing the non-significant path from Pittsburgh Sleep Quality Index to functional exercise adherence (*P* = 0.357), the revised model showed excellent fit: *χ*²/df = 0.842 (*P* = 0.359), RMSEA = 0.000, GFI = 0.992, NFI = 0.980, CFI = 1.000. Bootstrap analysis (1,000 samples) confirmed the robustness of all significant paths, with the bias-corrected 95% confidence intervals excluding zero (**Figure 2**).

Path analysis using standardized coefficients revealed that fear of cancer recurrence had a significant direct negative effect on quality of life (*β* = −0.920, *P* < 0.001) and a significant indirect effect mediated by reduced functional exercise adherence (*β* = −0.190, *P* = 0.011). The total effect of fear of cancer recurrence on quality of life was *β* = −1.086. The Pittsburgh Sleep Quality Index had a significant direct negative effect on quality of life (*β* = −1.139, *P* = 0.003), whereas functional exercise adherence had a marginally significant direct positive effect on quality of life (*β* = 0.874, *P* = 0.052) (**Table 5**).

**Table 5:** Pittsburgh sleep index and functional exercise adherence and fear of cancer recurrence path analysis of quality of life (standardized estimates).

Variable	Direct effect (95% CI)	Indirect effects (95% CI)	The total effect (95% CI)
Pittsburgh Sleep Index	-1.139 (-1.892, -0.386)	–	-1.139 (-1.892, -0.386)
Functional exercise adherence	0.874 (0.001, 1.749)	–	0.874 (0.001, 1.749)
Fear of cancer recurrence	-0.920 (-1.342, -0.498)	-0.166 (-0.324, -0.008)	-1.086 (-1.508, -0.664)

The model exhibited excellent fit indices; however, the RMSEA of 0.000 may indicate potential overfitting, as will be discussed subsequently.

## 4 Discussion

### 4.1 Trajectory of quality of life in postoperative breast cancer patients

Quality of life (QoL), a multidimensional indicator encompassing physiological, psychological, and social functioning, is a key outcome for evaluating breast cancer rehabilitation. This study found QoL scores of 87.44 ± 11.32 pre-discharge (3 to 4 days post-surgery), 104.16 ± 8.94 at 1 month, and 100.5 ± 11.02 at 3 months. Although a slight decline occurred relative to the 1-month peak, QoL at 3 months remained higher than the pre-discharge levels, indicating an overall trend of gradual improvement. As supported by Zhang et al. (21), significant functional recovery and adaptation to surgical trauma were observed after 1 month. With the passage of time, patients resumed daily life with reduced psychological stress, which contributed to the sustained improvement of QoL. Although a minor decrease occurred at 3 months (possibly related to work and lifestyle transitions), QoL remained substantially improved compared to the early postoperative phase. These findings suggest that early postoperative care should prioritize physical rehabilitation, with the aim of facilitating the restoration of bodily functions in a timely manner, while later stages require psychosocial support to promote social reintegration and sustained QoL improvement.

### 4.2 Postoperative functional exercise adherence, sleep, and recurrence of cancer fear of change among breast cancer patients of different stages

This study shows breast cancer patients with postoperative functional exercise adherence, sleep quality, and fear of cancer recurrence in a dynamic change (22). Exercise adherence was low in the early postoperative period, which may be attributed to surgical pain, fatigue, and treatment-related adverse effects. As rehabilitation progressed, exercise adherence improved significantly; this finding is inconsistent with that of Chu et al. (23), which may be due to the implementation of individualized rehabilitation programs and regular follow-up in their study. Sleep quality followed a fluctuating pattern: lowest at T1, improved at T2, and mildly declined again at T3. The initial decrease may relate to chemotherapy’s adverse effects, while T2 improvement may reflect psychological adaptation. The T3 rebound may be associated with fear of recurrence and long-term stress, consistent with the results of Lu et al. (24). Fear of cancer recurrence after surgery was the first to fall fast, within 3 months after the slow decline of linear trajectory. This is consistent with the research findings of Dunn et al. (25). Early postoperative recurrence in patients with fear is relatively strong, which may be related to the uncertainty of the surgical trauma and disease prognosis. As time goes by, the patients gradually accept their disease status and adapt to daily life, leading to a significant reduction in fear of cancer recurrence. Postoperative recovery is characterized by early physiological limitations, mid-term psychological and sleep improvements, and long-term stabilization. Clinical interventions should be stage-specific: early emphasis on pain management and exercise guidance, mid-term integration of psychological support and sleep interventions, long-term focus on maintaining healthy behaviors, and managing fear of recurrence to optimize a holistic recovery.

### 4.3 Analysis of influencing factors of quality of life of breast cancer patients

The results indicated that age, BMI, occupational status, daily working hours, daily gait speed, exercise intensity, and exercise time were significant factors influencing quality of life (QoL) in breast cancer patients. Specifically, elderly patients had lower QoL, which is consistent with the findings of Li et al. (26). Possibly due to decreased physical function and higher comorbidity burden, highlighting the need for enhanced health management and psychological support in this group. Higher BMI was also associated with reduced QoL, aligning with Carolyn et al. (27). Obesity may increase body burden and treatment side effects, underscoring the importance of weight management interventions (28). Occupational status and longer daily working hours significantly affected QoL, likely reflecting the dual burden of work and health management. The work-related stress and overall life burden of younger patients are higher, and related research results (29) indicate that low working pressure helps reduce psychological burden and promote rehabilitation. In addition, patients with a faster daily pace, higher intensity, and greater levels of activity tend to maintain a higher quality of life for a longer time, according to research results such as that of Burse et al. (30). Exercise can improve physical function and reduce treatment-related adverse effects. Thus, it is recommended to integrate exercise interventions into the routine postoperative rehabilitation program of breast cancer patients.

### 4.4 Exercise, sleep, and psychological fear jointly affect the quality of life of breast cancer patients

Path analysis revealed complex longitudinal associations among exercise, sleep, psychological fear (FCR), and QoL in breast cancer patients. While the temporal sequence provides evidence for potential mechanisms, the observational nature of our design necessitates a cautious interpretation of these relationships as associative rather than strictly causal.

Sleep quality, psychological state, and exercise engagement interact through multiple, interrelated pathways. Poor sleep contributes to daytime fatigue and reduced energy, which directly limits the patients’ exercise participation (31, 32). Similarly, fear of recurrence can lead to kinesiophobia, where patients avoid physical activity due to unwarranted safety concerns, further reducing exercise adherence (33). Both sleep disturbances and psychological distress also diminish self-efficacy, impairing their motivation to engage in regular exercise (34, 35).

These behavioral interactions are likely supported by underlying biological pathways. On a neurobiological level, regular exercise is known to promote the release of endorphins and reduce cortisol levels (36), which can alleviate psychological stress and improve mood. Improved mood and reduced stress, in turn, can lower hyperarousal and facilitate better sleep quality (37). Conversely, fear and poor sleep can dysregulate the hypothalamic–pituitary–adrenal (HPA) axis, increasing inflammation and fatigue (38). This physiological state creates significant biological barriers to exercise participation, forming a vicious cycle that ultimately impairs the overall quality of life.

Consequently, functional exercise compliance was positively associated with QoL, likely through mechanisms such as enhancing physical function and immunity, a finding consistent with prior research (30). The Pittsburgh Sleep Quality Index was negatively associated with QoL; sleep disorders may exacerbate fatigue, pain, and mood disturbances, potentially disrupting neuroendocrine and immune function (31, 32). Fear of recurrence adversely affected QoL through dual pathways: it directly impaired coping ability (33) and indirectly reduced QoL by decreasing exercise adherence and disrupting sleep patterns (34, 39).

Several important limitations of this study must be considered when interpreting these findings. First, the relatively small sample size (*n* = 50) from a single center limits the statistical power and generalizability of our results. Although we employed bootstrap resampling to enhance the robustness of our path estimates, the modest sample size may still affect the stability and precision of the structural equation model. The use of convenience sampling may also introduce selection bias. Second, although we employed a longitudinal design, the presence of unmeasured confounding variables cannot be ruled out. Furthermore, the excellent model fit (RMSEA = 0.000) should be interpreted with caution, as it may reflect overfitting to our specific sample rather than true population dynamics. Finally, the use of self-reported measures, though validated, may be subject to reporting bias. These limitations highlight the need for cautious interpretation and further validation in larger, more diverse populations.

In summary, the findings of this exploratory study are consistent with a sequential pathway wherein improved sleep may enhance psychological well-being, which could facilitate exercise adherence, and ultimately contribute to improved QoL (35). Specifically, better sleep alleviates symptoms of anxiety and depression while also strengthening psychological resilience (35). Better psychological health can enhance self-efficacy, encouraging participation in functional exercise (35). In turn, regular exercise not only improves physical function but also reduces psychological stress through endorphin release and other mechanisms, establishing a virtuous cycle. Clinically, exercise therapy might play a central role by improving physical function, reducing stress, and enhancing sleep, thereby disrupting the pathological cycle (40). Combined psychological and exercise interventions have been shown to improve sleep and reduce fear (41). Based on our preliminary findings, we suggest that postoperative management could consider incorporating exercise, sleep regulation, and psychological support into multidimensional rehabilitation strategies as potential avenues to holistically improve QoL.

## 5 Conclusion

This preliminary longitudinal study analyzed the changes in postoperative quality of life, functional exercise compliance, sleep quality, and fear of cancer recurrence among breast cancer patients, revealing a dynamic recovery process and identifying potential influencing factors of postoperative recovery. Quality of life initially improved but later declined, while functional exercise adherence, sleep quality, and fear levels varied significantly across time points. Age, occupational status, and exercise behavior were significant predictors of quality of life. Path analysis indicated that psychological fear had a significant inhibitory effect on exercise behavior in the early postoperative period, whereas the synergistic effects of exercise and sleep on QoL were gradually enhanced in the middle and late postoperative periods. Based on these preliminarily observed associations, a staged intervention strategy is tentatively proposed and warrants future empirical testing: 1 week after surgery (acute phase): pain management and mild joint movement were the main interventions, combined with cognitive behavioral therapy to alleviate fear; 1 month post-surgery (recovery phase): gradually increase the intensity of resistance training and simultaneously conduct sleep hygiene education to improve sleep quality; 3 months post-surgery (maintenance phase): strengthen home-based rehabilitation through community linkage and implement regular psychological assessments to prevent the rebound of fear of recurrence. For older patients (≥60 years) who may face greater functional limitations, exercise programs should prioritize safety and adherence. Therefore, the model and interpretations presented here should be viewed as preliminary and require validation in larger, more diverse cohorts. This study has several limitations. The sample was relatively small (*n* = 50) and drawn from a single institution, which may affect statistical power and generalizability. Convenience sampling could introduce selection bias, and self-reported measures, though validated, may be influenced by reporting bias. Although the model demonstrated excellent fit (RMSEA = 0.000), this perfect fit suggests potential overfitting, which may limit reproducibility in other populations. Finally, the 3-month follow-up might not fully capture long-term recovery trends. Future research should conduct multi-center studies with larger sample sizes and longer observation periods to validate the proposed theoretical model and intervention strategies.

## Data Availability

The raw data supporting the conclusions of this article will be made available by the authors, without undue reservation.
